# Editorial: The roles of immune cell homeostasis in cancer research and therapeutic response

**DOI:** 10.3389/fphar.2023.1227996

**Published:** 2023-06-21

**Authors:** Kui Zhang, Abhimanyu Thakur, Zhijie Xu, Zhi-Yao He

**Affiliations:** ^1^ State Key Laboratory of Resource Insects, Medical Research Institute, Southwest University, Chongqing, China; ^2^ The Pritzker School of Molecular Engineering, Ben May Department for Cancer Research, University of Chicago, Chicago, IL, United States; ^3^ Department of Pathology, Xiangya Hospital, Central South University, Changsha, Hunan, China; ^4^ Department of Pharmacy, West China Hospital, Sichuan University, Chengdu, Sichuan, China

**Keywords:** tumor microenvironment (TME), immune cell homeostasis, cancer pathogenesis, therapeutic response, tumor-immune cell interaction, cancer-associated molecules, immune cell-based therapeutic targets

Immune cells within the tumor microenvironment (TME) significantly contribute to the composition and dynamics of the TME, thereby influencing cancer pathogenesis and therapeutic response. The dynamic balance of interactions among immune cells and between immune cells and tumor cells has been identified as a promising therapeutic strategy for treating various cancers. Several therapies have been demonstrated to alleviate such immune disorders and restore immune cell homeostasis. Therefore, it is urgently necessary to deeply explore the significant implications of immune cell homeostasis in cancers, and to unravel the underlying molecular mechanisms and biological functions of this interplay, with the goal of improving treatment efficacy.

This Research Topic aims to explore the Frontier research in the context of immune cell homeostasis in the TME, with a spotlight on 1) immune cell-based strategies for cancer research and treatment; 2) the interaction between cancer cells and immune cells; 3) the molecular mechanisms of tumor-infiltrating immune cell regulation; 4) underlying roles of cancer-associated molecules in immune cell infiltration; 5) clinical or bioinformatics analyses to explore immune cell-based therapeutic targets.

Huang et al. focused their research on the integration of artificial intelligence with immunosignals for the precision treatment of liver diseases associated with Hepatitis B Virus (HBV). They identified CLST and aCD4, two immunosignals integral to the virus’s pathogenesis, as key players in the inflammation, fibrosis, and hepatocellular carcinoma triggered by HBV. Using gene set variation analysis, they developed immunogenomic signatures that streamlined the creation of robust diagnostic and prognostic models. The clinical application of CLST and aCD4 as indicators could significantly improve the precision management of hepatocellular carcinoma. This study provides an in-depth understanding of the gene characteristics tied to the immune microenvironment in HBV infection and offers subtle insights for the clinical management of HBV-related hepatocellular carcinoma, thereby establishing a strong foundation for precision medicine. Liu et al. successfully integrated machine learning to build classifiers related to aberrant alternative splicing (AS) events. These classifiers are designed to predict prognosis and the response to immunotherapy in patients with hepatocellular carcinoma. They found that AS can be instrumental in classifying HCC subtypes, as it alters the activity of tumor-related pathways through differential splicing effects. This, in turn, impacts the TME and plays a role in immune reprogramming. The authors have outlined the clinical and molecular characteristics, offering a fresh approach for personalized treatment of HCC patients.

Glioma is one of the most common types of primary brain tumor. Li et al. reveal that the transcription factor ZBTB42 is highly expressed in glioma, it could be regarded as a promising prognostic factor for glioma. Moreover, ZBTB42 appears to be linked to immune cell infiltration, potentially playing a role in the immune-suppressive TME. Notably, the study also found a correlation between ZBTB42 and stem cell markers, indicating a positive association with glioma stemness. Therefore, the research identifies ZBTB42 as a prognostic biomarker for glioma, with its function tied to both the suppressive TME and the stemness of glioma. Fang et al. found that URB2 is also significantly overexpressed in glioma, and has a potential oncogenic role, as evidenced by the substantial impairment of cell viability upon its knockdown. Its expression level can independently predict overall survival. Interestingly, a strong link between URB2 and immune responses has been discovered, with the URB2 phenotype possibly contributing to immune suppression in GBM. This study indicated URB2 may serve as a crucial tool for prognosis prediction and immunotherapy guidance in glioma treatment. Luo et al. conducted a bioinformatic analysis of IL-15, a cytokine with diverse roles in immune regulation and tumorigenesis, as a potential prognostic biomarker across various cancers and its link to exercise’s anti-cancer effects. They discovered that IL-15 is generally downregulated in most cancers, with its high expression predicting better survival outcomes. Amplification emerged as the most common mutation type in IL-15’s genome. Additionally, IL-15 expression correlated with the infiltration levels of different immune cells and positively associated with ferroptosis/cuproptosis-related genes (ACSL4 and LIPT1) across various cancers. This study underscores IL-15’s potential as a prognostic biomarker for patient outcomes, immune responses, and ferroptosis/cuproptosis in pan-cancer, shedding light on exercise’s anti-cancer effects.

Li et al. found that tissues resistant to TPF chemotherapy, a regimen comprising Docetaxel, Cisplatin, and Fluorouracil, in HPSCC patients exhibited upregulated T cell activation and downregulated glycolysis. They identified SEC61G as a key gene negatively correlated with CD8^+^ T cells and involved in glycolysis. Their findings suggest that while enhanced glycolysis may promote immune escape, it may also increase TPF chemotherapy response. Targeting the E2F1/SEC61G pathway could potentially boost MHC-I expression, offering a new therapeutic avenue.

Zeng et al. analyzed the link between TGF-β signaling pathway-related genes (TSRGs), clinical prognosis, the TME, and immunotherapy in gastric cancer. This study discerned two unique TGF-β subgroups in gastric cancer, with one subgroup showing an immunosuppressive environment and reduced survival. A new TGF-β-related prognostic model was developed, indicating that patients with lower risk scores have improved prognosis and are more responsive to immunotherapy. These insights emphasize the role of TSRGs in shaping the tumor immune microenvironment and tailoring immunotherapy for gastric cancer patients. Interestingly, TGF-β has also been explored as a potential prognostic biomarker for glioma. Chen et al. have identified a crucial connection between serine and glycine metabolism-related genes (SGMGs) and both the prognosis and immune microenvironment of glioma. They’ve constructed a unique SGMG signature that holds promise in predicting patient prognosis and immune responses. This research implies that SGMGs could potentially steer the choice of immunotherapy in treating glioma. Qi et al. analyzed the genomic and transcriptomic profiles of 34 anoikis-related genes (ARGs), which are crucial for maintaining immune cell balance. The researchers found significant differences in ARG expression between soft-tissue sarcoma and normal tissues, suggesting a potential disruption of immune homeostasis in cancer. Their anoikis scoring system, which effectively predicted immune cell infiltration and immunotherapy response, could serve as a tool for assessing immune status and guiding personalized immunotherapy in cancer treatment. Meng et al. identified two molecular subtypes in uveal melanoma (UM) based on matrix-remodeling associated genes (MAGs), which showed significant differences in clinical outcomes. They developed a risk score system involving six MAGs that effectively predicted prognosis and immune activity. Their findings suggest that this MAGs-based system could enhance prognosis assessment and guide clinical decision-making in UM, highlighting the role of immune cell homeostasis in cancer therapy response.

Clearly, these research findings will serve as a critical source of information to benefit all stakeholders involved in understanding the impact of immune cell homeostasis in cancer and their potential therapeutic responses.

